# Efficacy between low and high dose aspirin for the initial treatment of Kawasaki disease: Current evidence based on a meta-analysis

**DOI:** 10.1371/journal.pone.0217274

**Published:** 2019-05-22

**Authors:** Xiaolan Zheng, Peng Yue, Lei Liu, Changqing Tang, Fan Ma, Yi Zhang, Chuan Wang, Hongyu Duan, Kaiyu Zhou, Yimin Hua, Gang Wu, Yifei Li

**Affiliations:** 1 Department of Pediatrics, West China Second University Hospital, Sichuan University, Chengdu, Sichuan, China; 2 Ministry of Education Key Laboratory of Women and Children's Diseases and Birth Defects, West China Second University Hospital, Sichuan University, Chengdu, Sichuan, China; 3 West China Medical School, Sichuan University, Chengdu, Sichuan, China; 4 Program for Changjiang Scholars and Innovative Research Team in University, West China Second University Hospital, Sichuan University, Chengdu, Sichuan, China; University of Mississippi Medical Center, UNITED STATES

## Abstract

**Background:**

Kawasaki disease (KD) is now the leading cause of acquired heart disease in children in developed countries. Intravenous immunoglobulin (IVIG) and aspirin were considered as the standard initial treatment of KD for decades. However, the optimal dose of aspirin has remained controversial. In recent years, many studies compared the efficacy of low-dose with high-dose aspirin in the acute phase of KD, but the results have not always been consistent. Therefore, we performed this meta-analysis to evaluate the efficacy of low-dose aspirin compared with high-dose for the initial treatment of KD.

**Methods:**

Studies related to aspirin therapy for KD were selected from PubMed, EMBASE, the Cochrane Central Register of Controlled Trials, China National Knowledge Infrastructure, and Google scholar through Mar 25^th^, 2019. Data were analyzed using STATA Version 15.1. Additionally, publication bias and sensitivity analysis were also performed by STATA version 15.1.

**Results:**

Six studies were included in our analysis of the rate of coronary artery lesion (CAL), five reports for IVIG-resistant KD (rKD), and four for the duration of fever and hospitalization. However, no significant differences were found between low-dose and high-dose aspirin groups in the incidence of CAL (risk ratio (RR), 0.85; 95%CI (0.63, 1.14); P = 0.28), the risk of rKD (RR, 1.39; 95%CI (1.00, 1.93); P = 0.05), and duration of fever and hospitalization (the mean standard deviation (SMD), 0.03; 95%CI (-0.16, 0.22); P = 0.78).

**Conclusion:**

Low-dose aspirin (3–5 mg·kg^-1^·d^-1^) may be as effective as the use of high-dose aspirin (≥30 mg·kg^-1^·d^-1^) for the initial treatment of KD. Further well-designed randomized clinical trials are needed to evaluate the efficacy of low-dose aspirin for the initial treatment of KD.

## Introduction

Kawasaki disease (KD) is an acute, self-limited febrile vasculitis of unknown cause that predominantly affects children under five years of age [1]. KD is now the most common cause of acquired heart disease in children in developed countries [2]. In general, KD is regarded as an innate immune disorder resulting from the exposure of a genetically predisposed individual to microbe-derived innate immune stimulants [3]. However, the etiology and pathogenesis of KD are still unclear. In addition, coronary artery aneurysm (CAA) is a severe cardiovascular complication of KD, and timely initiation of treatment with intravenous immunoglobulin (IVIG) has reduced the incidence of CAA from 25% to ≈4% [4]. Moreover, previous studies found that thrombocytosis is universal in the subacute stage of KD [5–7]. Furthermore, the degree of platelet activation was closely associated with the presence of coronary artery complications in the acute stage of KD [7]. Therefore, anti-inflammatory and anti-platelet therapies are the primary treatments for KD.

Aspirin, or acetylsalicylic acid (ASA), was first synthesized in 1897 and has been used as a pain reliever in some form dating back to ancient Egypt [8]. Both the beneficial and harmful effects of aspirin are thought to be primarily due to inhibition of prostanoid biosynthesis, particularly thromboxane A_2_ (TXA_2_) and prostaglandins (e.g., PGE_2_ and PGI_2_) [9]. Aspirin irreversibly inhibits cyclooxygenase 1 (COX-1) by acetylation of the amino acid serine at position 529 [10], thereby preventing arachidonic acid access to the COX-1 catalytic site through steric hindrance. By inhibiting COX-1, platelets cannot synthesize prostaglandin H2, which is normally converted to TXA_2_ via the enzyme thromboxane synthase [11]. COX-2 is the second cyclooxygenase isoenzyme, and is primarily responsible for the synthesis of the platelet inhibitor PGI_2_ by endothelial cells [12]. COX-2 is induced in response to inflammatory stimuli and is less sensitive to the effects of aspirin. Moreover, aspirin is 170-fold less effective at inhibiting COX-2 than COX-1 [13]. Therefore, high-dose aspirin is mainly used for anti-inflammatory therapy, while low-dose aspirin is used for antiplatelet therapy.

The American Heart Association (AHA) recommends that the standard treatment regimen for the acute phase of KD involves administering IVIG 2 g·kg^-1^ within ten days of onset and aspirin moderate (30–50 mg·kg^-1^·d^-1^) to high-dose (80–100 mg·kg^-1^·d^-1^) until the patient is afebrile [14]. This acute phase regimen is designed primarily for anti-inflammatory treatment. However, side effects including gastritis, upper gastrointestinal bleeding [15], anemia [16], and Reye’s syndrome [17] have been reported in KD children receiving high-dose aspirin treatment. Previous studies have shown that the incidence of coronary artery lesions (CAL) is highly dependent on the dosage and infusion timing of IVIG, but not related to the aspirin dose [18,19]. These results suggest that the efficacy of low-dose aspirin may be adequate for the initial treatment of KD, and can also reduce the risk of complications caused by high-dose aspirin. According to this, we generated this meta-analysis followed PICO (patient problem or population, intervention, comparison or control, and outcome) principle. The population was focused on Kawasaki patients, and we evaluated the intervention of aspirin administration. The comparison had been made among a series of dosages of aspirin. Outcome measurement had been selected to demonstrate the efficacy of different dosages of aspirin. To explore this problem, researchers have recently performed many related studies [20–22], but the results have not always been consistent. Therefore, we performed this meta-analysis to evaluate whether low-dose aspirin was as effective as high-dose aspirin for the initial treatment of KD.

## Materials and methods

### Study protocol

This analysis was performed following a predetermined protocol by the recommendations of the Cochrane Handbook of Systematic Reviews. The data collection and reporting were in accordance with the Preferred Reporting Items for Systematic Reviews and Meta-Analyses (PRISMA) Statement (S1 Table) [23].

### Search strategy

We searched multiple databases including PubMed, EMBASE, the Cochrane Central Register of Controlled Trials, China National Knowledge Infrastructure, and Google scholar through Mar 25th, 2019 to identify relevant studies. Keyword search terms were (‘mucocutaneous lymph node syndrome’ OR ‘Kawasaki disease’ OR ‘Kawasaki syndrome’) AND (‘aspirin’ OR ‘acetylsalicylic acid’ OR ‘salicylate’). We searched the PubMed database as follows: (Mucocutaneous Lymph Node Syndrome[MeSH Terms] OR Kawasaki disease OR Kawasaki syndrome) AND (aspirin[MeSH Terms] OR acetylsalicylic acid OR salicylate). Search terms for EMBASE, the Cochrane Central Register of Controlled Trials, China National Knowledge Infrastructure, and Google scholar with corresponding publication numbers can be found in the S1 Appendix. Languages were limited to English and Chinese.

### Study selection

We initially screened studies by title and abstract for the systematic search. A full-text search that retrieved potentially relevant reports was assessed for compliance using the inclusion and exclusion criteria. Inter-rater reliability for the study selection was calculated using the kappa statistic.

#### Criteria for inclusion

1) All subjects were patients diagnosed with KD; 2) randomized controlled or non-randomized controlled clinical trials or cohort studies evaluating the efficacy of low-dose (3–5 mg·kg^-1^·d^-1^) and high-dose (≥30 mg·kg^-1^·d^-1^) aspirin combined with standard IVIG (2 g·kg^-1^) therapy for the initial treatment of KD; 3) outcome interests were as follows: the incidence of CAL included CAA, coronary artery dilatation or ectasia, days of fever or hospital, the rate of IVIG-resistant KD (rKD), and the presence of adverse events.

#### Criteria for exclusion

Studies meeting any of the following criteria were excluded: 1) patients were not treated with standard IVIG (2 g·kg^-1^); 2) patients lacked a low-dose or high-dose aspirin group for the initial treatment of KD; 3) conference articles, reviews, or abstracts; 4) duplicate reports.

### Data collection and assessment of study quality

Two investigators (Xiaolan Zheng and Peng Yue) independently assessed the eligibility of reports at the title and abstract level, and a third reviewer (Yifei Li) determining divergence according to the inclusion or exclusion criteria and the quality of the reports. Studies that met all inclusion criteria were selected for further analysis. The baseline data from the included studies were extracted and shown in Table 1. The quality of the included nonunionized studies in meta-analyses was assessed using the Newcastle-Ottawa Scale (NOS) [24]. Additionally, studies which scored at least five stars were considered to have moderate to high methodological quality.

**Table 1:** Characteristics of involved KD patients in all included studies.

First author(ref.)	Year	Country	Follow-up(weeks)	Specific dosage of high-dose aspirin group(mg·Kg^-1^·d^-1^)	Study type	Age (years)	Male (%)	Patients no.	Illness day at enrolment	CRP (mg L^-1^)	WBC (×10^9^ L^−1^)	Hb (g L^-1^)	PLT (×10^9^ L^−1^)	NOS
Rahbarimanesh[15]	2014	Iran	8–10	80–100	Retrospective	N/R	N/R	42/27	N/R	N/R	N/R	N/R	N/R	5
Kim[16]	2017	Korea	13	≥30	Retrospective	2.6/2.7	57.4/58.3	509/7947	N/R	89.9/92.3	14.1/13.8	113/115	347.2/349.0	7
Dhanrajani[17]	2017	Canada	6–8	80–100	Retrospective	2.7/3	59/58.3	122/127	6.5/6	N/R	N/R	N/R	N/R	6
Dallaire[18]	2017	Canada	52	80–100	Retrospective	3.2/3.4	58.4/59.4	365/848	6.2/6.2	100.6/78.6	14.7/13.6	109.4/113.8	368.9/370.3	7
Amarilyo[19]	2017	Israel	N/R	80–100	Retrospective	2.9/2.4	65.1/63.5	43/315	9.1/8.1	N/R	N/R	N/R	N/R	6
Huang[12]	2018	China	104	30–50	Retrospective	2.0/2.1	63/69	672/86	N/R	76.0/105.8	15.4/17.9	N/R	381.9/382.3	7

CRP C-reactive protein, WBC white blood cell, Hb hemoglobin, PLT platelet count, NOS Newcastle-Ottawa scale, N/R not report.

### Outcome measures

The primary outcome was the incidence of CAL, including CAA, coronary artery dilatation or ectasia. Secondary outcomes included rKD, days of hospital or fever, and adverse events. The potential adverse events for aspirin were allergies, digestive symptoms (diarrhea, vomiting and gastric ulcer), abnormal liver function and hemorrhage (skin petechia, epistaxis) [25].

### Publication bias

Publication bias was tested using Egger’s regression by Stata statistical software (STATA) version 15.1. Each dot represents each study in the meta-analysis. Asymmetry of the dot distribution between regression lines indicates potential publication bias. A quantified result of P < 0.05 in Egger’s test indicated that publication bias existed [26].

### Heterogeneity

Heterogeneity in the pooling sensitivity and specificity was examined using the Chi-squared test and was deemed to be statistically significant when P < 0.05 in these qualitative tests. The I^2^ test was also performed in every pooling analysis to quantitatively estimate the proportion of total variation across studies that were due to heterogeneity rather than chance. The I^2^ value can range from 0 to 100%, and a value greater than 50% suggests significant heterogeneity.

### Meta-regression and sensitivity analysis

We performed the meta-regression to detect where the potential factor for heterogeneity originated. Sensitivity analysis was conducted for every study to determine whether any single study incurred undue weight in the analysis using STATA 15.1 for meta-analysis fixed/random-effects estimates.

### Statistical analysis

Data analysis was performed with STATA 15.1 [27]. The numbers of patients who developed CAL and rKD in each study were converted to risk ratio (RR). Days of fever or hospital were expressed with mean ± standard deviation (mean ± SD). Homogenous results utilized the fixed effects model (M-H method) for statistical analysis, while heterogeneous (I^2^ > 50%) results utilized the random effects model (M-H method) and were presented using a forest map. Additionally, publication bias and sensitivity analysis were also performed with STATA 15.1.

## Results

### Search results

**Fig 1:**
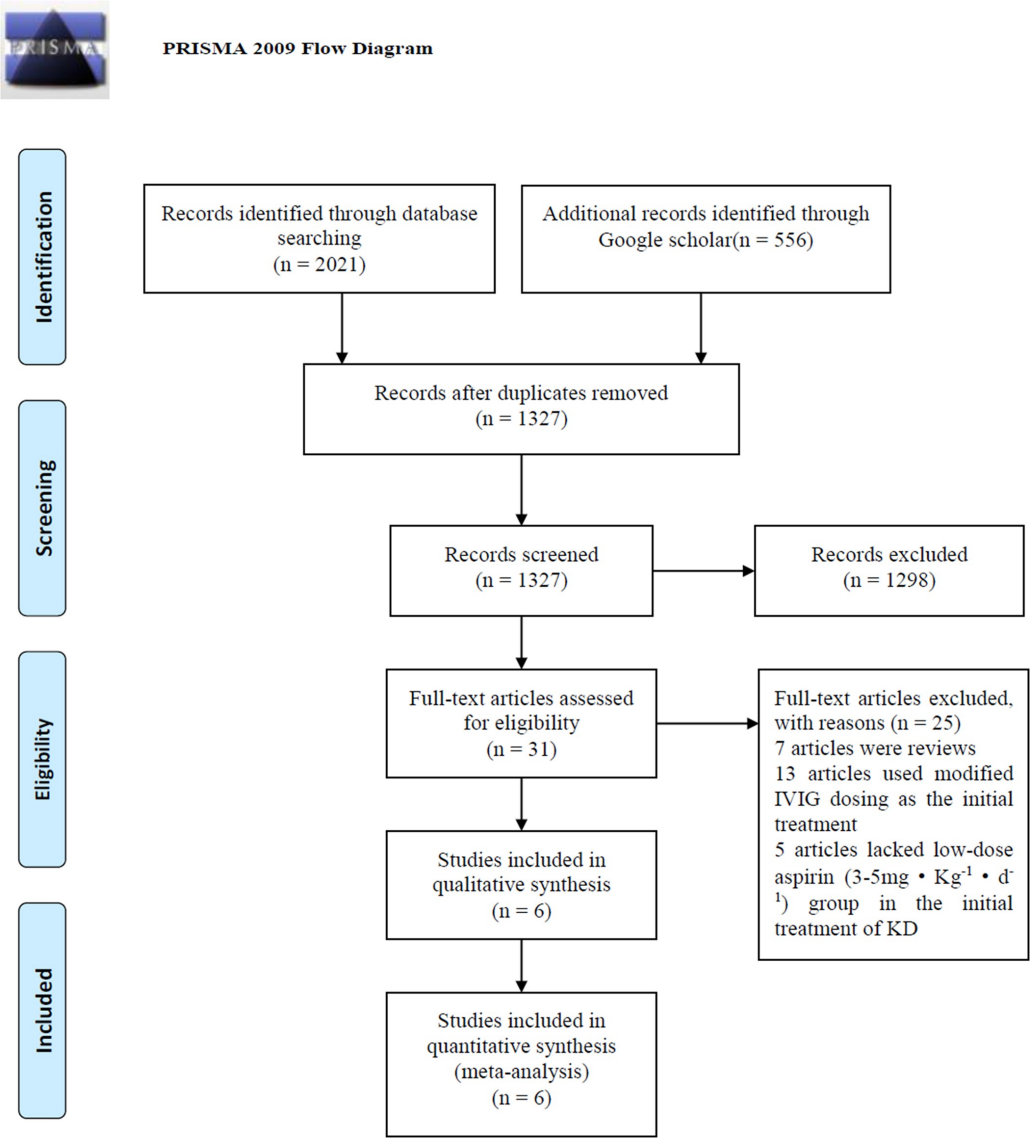
PRISMA flow chart of studies inclusion and exclusion. From: Moher D, Liberati A, Tetzlaff J, Altman DG, The PRISMA Group (2009). Preferred Reporting Items for Systematic Reviews and Meta-Analyses: The PRISMA Statement. PLoS Med. 6(7): e1000097. doi:10.1371/journal.pmed.1000097. For more information, visit www.prisma-statement.org.

Initially, 2577 potentially relevant papers were retrieved by the aforementioned search method, of which, 31 articles were considered to be of interest after reading titles and abstracts. However, 25 articles were excluded by reading the complete articles due to article type (n = 7), not using the standard IVIG dose (2 g·kg^-1^) during the initial treatment (n = 13), and lacking a low-dose aspirin (3–5 mg·kg^-1^·d^-1^) group for the initial treatment of KD (n = 5). Ultimately, six English reports [20–22,25,28,29] were included in the meta-analysis, while no Chinese reports met the inclusion criteria. The process of study selection was illustrated in Fig 1. The kappa test showed that the kappa value of agreement during the systematic searches was 0.83.

### Study characteristics

The characteristics of the involved patients in all included studies were presented in Table 1. The six published reports [20–22,25,28,29] enrolled a total of 11,103 children, of which were 1,753 low-dose aspirin cases. Six studies that were included came from five countries. All included studies were nonrandom and retrospective trials. Two studies [28,29] did not specify a criteria for dividing patients into a low-dose or high-dose aspirin group, three studies [20–22] grouped patients by different centers, and one study [25] chose different doses of aspirin depending on the inflammatory level and doctors’ opinions. Furthermore, three studies [21,28,29] did not mention the diagnostic criteria for KD, while another three reports [20,22,25] used the AHA diagnostic criteria [1]. Moreover, one study [28] scored NOS five stars and was considered to be moderate quality, while the other five reports were awarded ≥ six stars and qualified as high quality.

### Primary outcomes

#### Coronary artery lesion

**Fig 2:**
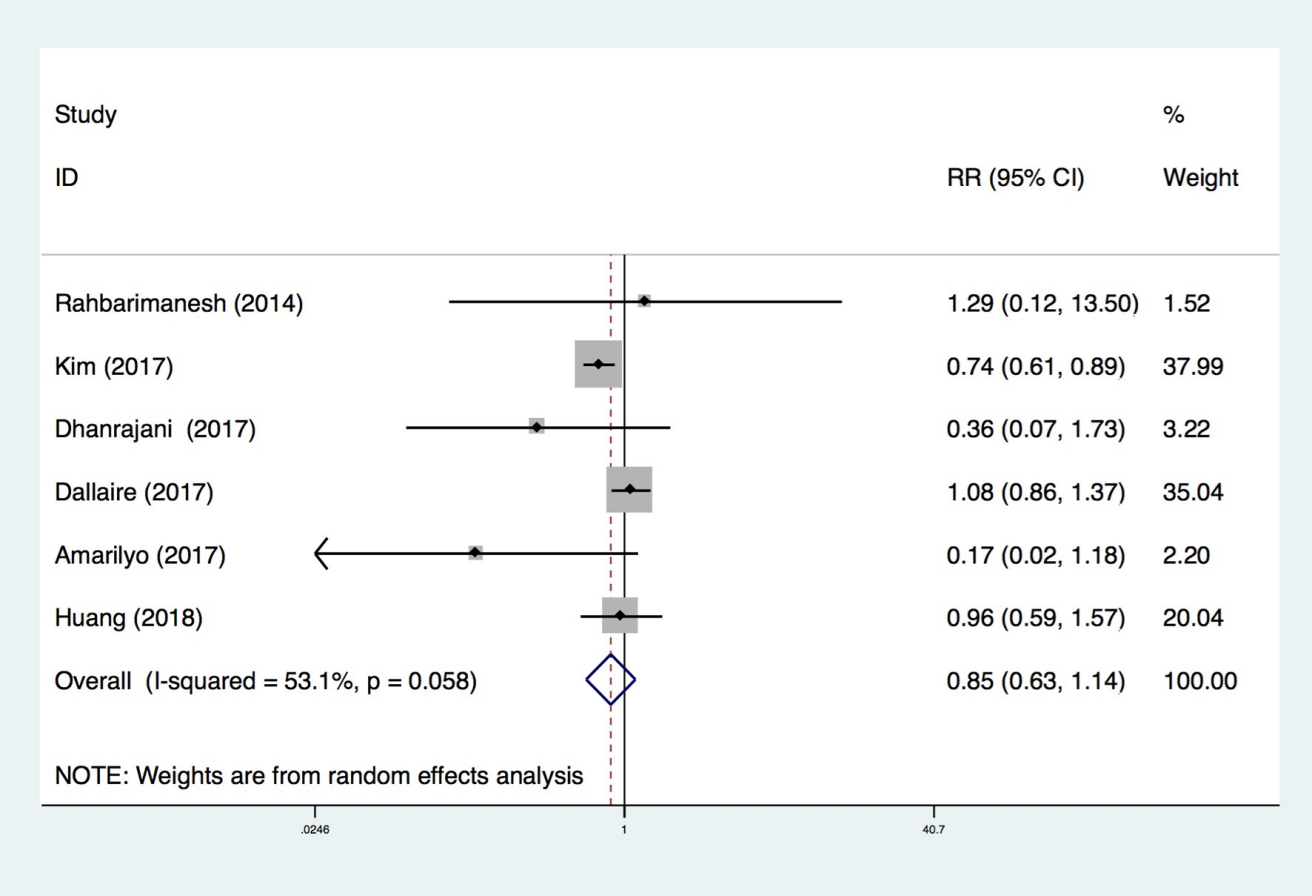
Forest plot for comparison of the incidence of coronary artery lesion identified in the meta-analysis of six trials using random-effect model. Only the first author of each study is given. Test for overall effect, z = 1.09, P = 0.28; test for heterogeneity, I^2^ = 53.1%, P = 0.058. RR, risk ratio. CI, confidence interval.

**Fig 3:**
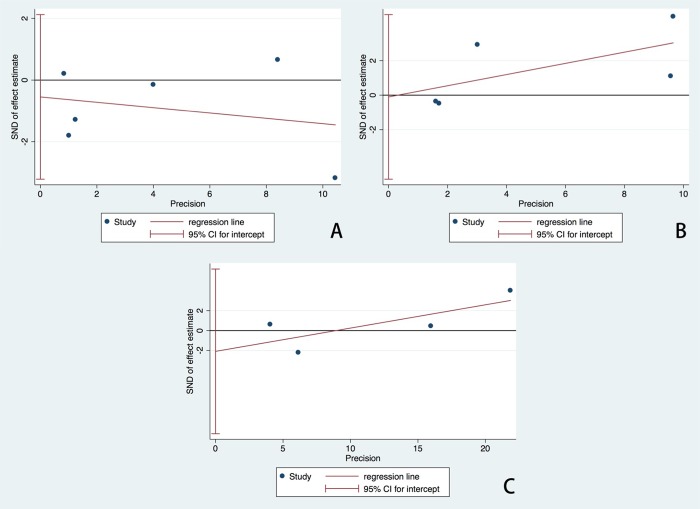
Egger’s publication bias plots for the assessment of potential publication bias. Each dot represents each study in the meta-analysis. Asymmetry of the dot distribution between regression lines indicates potential publication bias. (A) For the risk measurement of CAL, the Egger’s plot did not show marked asymmetry, P = 0.601, t = -0.57, 95% CI (-3.22, 2.12), (B) For the risk analysis of rKD, the Egger’s plot did not show significant asymmetry, P = 0.950, t = -0.07, 95% CI (-4.87, 4.66), (C) For the evaluation of days of fever or hospital, the Egger’s plot also did not show significant asymmetry, P = 0.390, t = -1.09, 95%CI (-10.37, 6.19). This Egger’s plot indicates no publication bias with a P value >0.05. RR, risk ratio. CI, confidence interval.

Although all trials reported the rate of CAL, the criteria of diagnosis for CAL varied. Three studies [20,22,28] did not mention the criteria they used, one study [21] used the AHA Z-score criteria [30], and one report [25] used the Japanese Cardiology Society (JCS) criteria [31]. One study [29] utilized both the AHA Z-score and JCS criteria to evaluate CAL. In such a situation, we preferred to pool the results according to AHA Z-score analysis in order to reveal the severity of coronary artery dilation adjusted by body surface area [14]. Overall, low-dose aspirin treatment did not increase the risk of CAL compared with the high-dose group (RR 0.85; 95%CI (0.63, 1.14); P = 0.28) (Fig 2). However, there was significant heterogeneity (I^2^ = 53.1%). The Egger’s publication bias plot did not show marked asymmetry (P = 0.601, t = -0.57, 95%CI (-3.22, 2.12)) (Fig 3A).

### Secondary outcome

#### IVIG resistance

**Fig 4:**
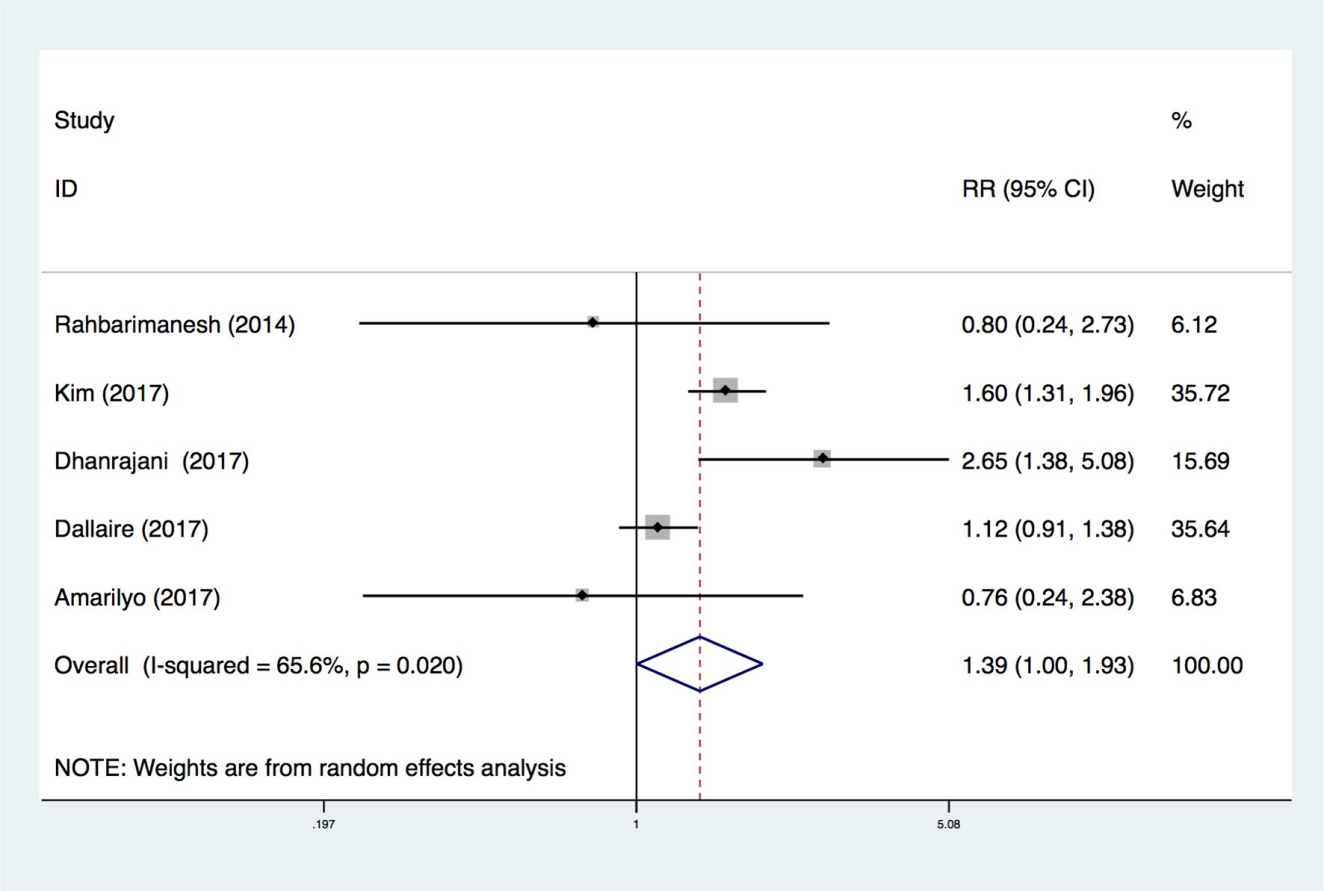
Forest plot for comparison of the rate of IVIG-resistant KD in the meta-analysis of five trials using random-effect model. Only the first author of each study is given. Test for overall effect, z = 1.98, P = 0.05; test for heterogeneity, I^2^ = 65.6%, P = 0.020. RR, risk ratio. CI, confidence interval.

Five studies provided data on the rate of IVIG resistance [20–22,28,29]. However, three [20,28,29] studies did not mention a diagnostic criteria for rKD, one study [21] defined patients with rKD as having the requirement for further treatment after the first dose of IVIG, and one report [22] defined patients with recurrence of fever after >72 hours as the rKD group. Overall, the risk of rKD in the low-dose group was not significantly different compared with the high-dose group (RR, 1.39; 95%CI (1.00, 1.93); P = 0.05) (Fig 4). Moreover, significant heterogeneity remained (I^2^ = 65.6%). The Egger’s publication bias plot did not show marked asymmetry (P = 0.950, t = -0.07, 95%CI (-4.87, 4.66)) (Fig 3B).

#### Days of fever or hospital

**Fig 5:**
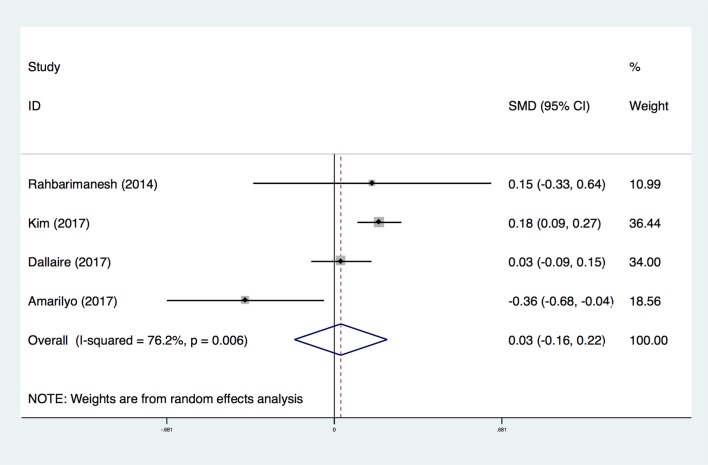
Forest plot for comparison of days of fever or hospital in the meta-analysis of four trials using random-effect model. Only the first author of each study is given. SMD, the mean standard deviation. Test for overall effect, z = 0.27, P = 0.78; test for heterogeneity, I^2^ = 76.2%, P = 0.006. RR, risk ratio. CI, confidence interval.

Four trials provided data about days of fever or hospital [21,22,28,29]. In total, there were no significant differences in the days of fever or hospital (SMD 0.03; 95%CI (-0.16, 0.22); P = 0.78) between groups (Fig 5). In addition, significant heterogeneity remained (I^2^ = 72.6%). The Egger’s publication bias plot did not show marked asymmetry (P = 0.390, t = -1.09, 95%CI (-10.37, 6.19)) (Fig 3C).

#### Adverse events

Four studies [20,21,28,29] in our meta-analysis did not report any adverse effects of aspirin. One study [22] reported three cases of epistaxis, rectal bleeding, and abdominal pain attributed to aspirin in the high-dose group, but there was no mention of the low-dose group. Another trial [25] reported that digestive symptoms were the most common side effects, and approximately 0.9% of patients had severe liver function damage, but there was no indication as to which group this occurred. Therefore, adverse events could not be summarized or analyzed based on this situation.

#### Meta-regression and sensitivity analysis

**Fig 6:**
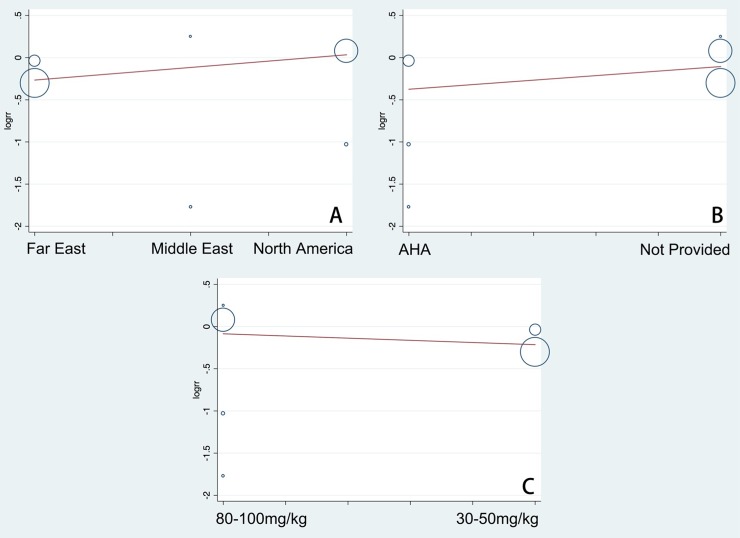
The meta-regression of the enrolled studies. (A) For the regions of studies, the meta-regression did not detect it is a dramatic impact on the homogeneity of the enrolled studies, P = 0.220, t = 1.45, 95%CI (0.87, 1.55). (B) For diagnostic criteria for KD, the meta-regression did not find it is a dramatic impact on the homogeneity of the enrolled studies, P = 0.542, t = 0.67, 95%CI (0.43, 4.02). (C) For specific dosages of high-dose aspirin groups the meta-regression did not detect it is a dramatic impact on the homogeneity of the enrolled studies, P = 0.713, t = -0.39, 95%CI (0.35, 2.19). The meta-regression could determine the correlation between the potential factors and the existing heterogeneities. When a significant difference was discovered, the factor should have a dramatic impact on the homogeneity of the enrolled studies with a P value >0.05. rr, risk ratio. CI, confidence interval.

**Fig 7:**
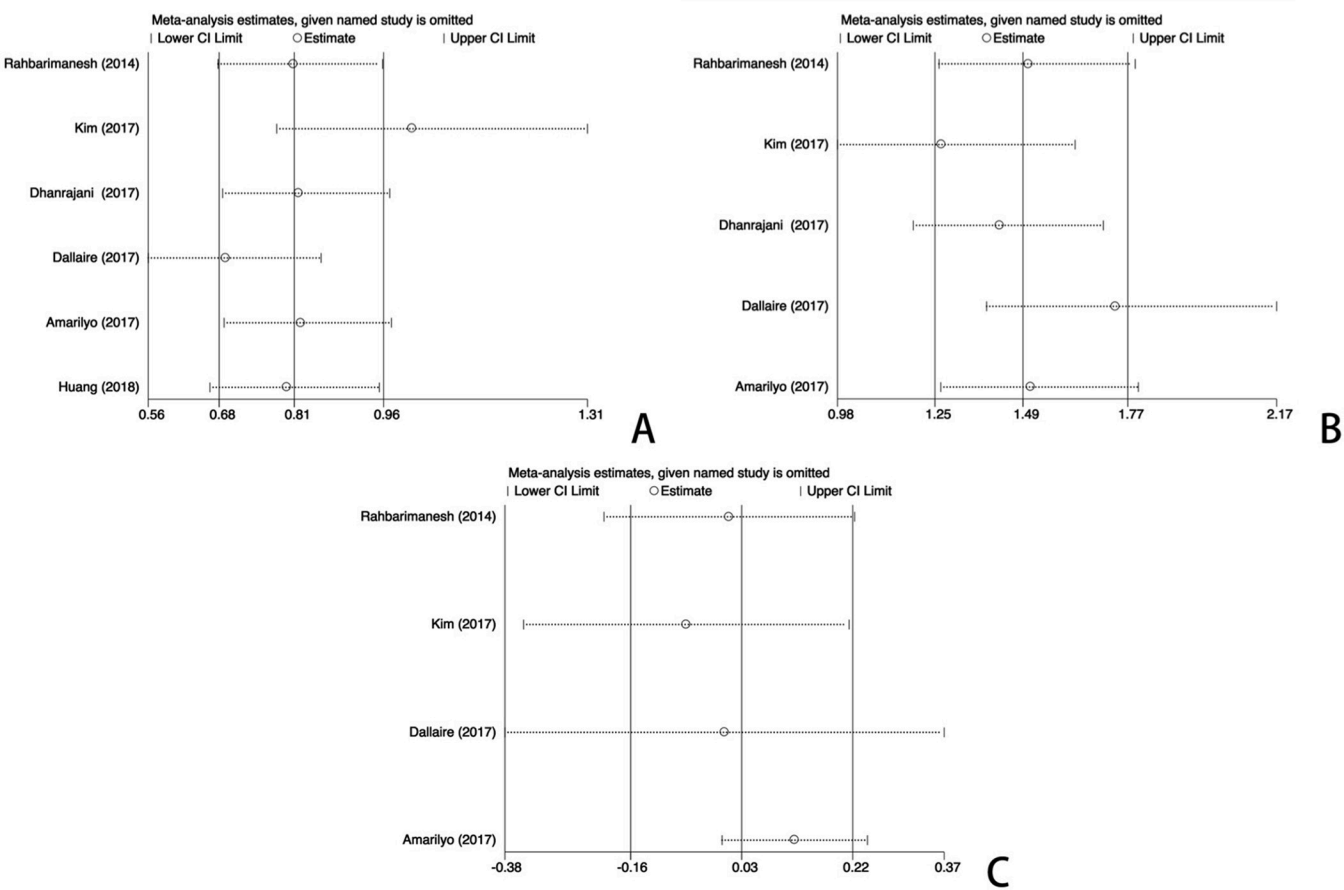
Sensitivity analysis of the individual trials on the results. (A) For the incidence analysis of CAL, (B) For the risk analysis of rKD, (C) For the evaluation of days of fever or hospital. Not any single study was detected to incur undue weight in the analysis.

We performed the meta-regression analysis to identify the potential factors that might cause heterogeneity. All potential factors that were extracted from the baseline measurements and original testing procedures were taken into consideration for the meta-regression. The meta-regression could determine the correlation between the potential factors and the existing heterogeneity. When a significant difference was discovered, the factor should have a dramatic impact on the homogeneity of the enrolled studies with a P value < 0.05. The study regions, the diagnostic criteria for KD, and the specific dosages of the high-dose aspirin groups were taken into account in the meta-regression to detect the origins of heterogeneity. According to the results (Fig 6), the meta-regression did not detect that the study regions had a dramatic impact on the homogeneity of the enrolled studies, P = 0.220, t = 1.45, 95%CI (0.87, 1.55) (Fig 6A). The meta-regression did not find that the diagnostic criteria for KD was a dramatic impact factor, P = 0.542, t = 0.67, 95%CI (0.43, 4.02) (Fig 6B). The meta-regression also did not detect that specific dosages from the high-dose aspirin groups had a dramatic impact on the homogeneity of the enrolled studies, P = 0.713, t = -0.39, 95%CI (0.35, 2.19) (Fig 6C). Therefore, different regions, different diagnostic criteria for KD, and the differences in specific dosage in the high-dose aspirin group were not responsible for the existing heterogeneity. Furthermore, we systematically and qualitatively analyzed the sensitivity across the included studies to determine the influence of individual trials on the results. Finally, we did not detect any significant impact from any single study and confirmed the direction of the results (Fig 7).

## Discussion

As the most common cause of acquired heart disease in children in many countries [2,32,33], the timeline of using aspirin in the acute phase of KD originated earlier than that of using single high-dose IVIG [34]. However, recent studies [35,21] indicated that the risk of CAL was not related to the aspirin dose, which depended only on the timing and dosage of IVIG. Furthermore, patients who received high-dose aspirin faced a higher risk of medication side effects, including dose-dependent minor bleeding events and gastric injuries [9]. Therefore, we conducted this meta-analysis and found no increased risk of CAL in the low-dose group compared with the high-dose group. This result suggested that high-dose aspirin administration aimed at reducing the risk of CAL may not be required in the acute phase of KD.

It is worth noting that the generally accepted diagnostic criteria for KD are the Japan Kawasaki Disease Research Committee (JKDRC) [36] and AHA diagnostic criteria [1], both of which are based on clinical features. However, the diagnosis of KD is still very challenging because its presentation can be quite varied [37]. In addition, the criteria in all studies for the diagnosis of KD, rKD, CAL, and for dividing patients into a low-dose or high-dose aspirin group differed. There were no randomized, controlled studies comparing only high-dose aspirin (>80 mg·kg^-1^·d^-1^) with moderate-dose aspirin (≤50 mg·kg^-1^·d^-1^) to resolve the signs and symptoms of inflammation in KD, which may bias the evaluation of high-dose aspirin's effectiveness at different dose ranges. Although the meta-regression analysis indicated that the different regions, different diagnostic criteria for KD, and the differences in the specific dosage of the high-dose aspirin group were not responsible for the existing heterogeneity, the differences in methodology may still cause certain bias. Therefore, further well-designed studies with uniform criteria are needed to evaluate the effect of low-dose aspirin for the initial treatment of KD.

Approximately 9.4% to 23% of patients with KD would have a persistent or recurrent fever after their first IVIG therapy [38]. Furthermore, rKD patients can suffer a 9-fold greater risk of developing CAA compared with non-rKD patients (12.2% vs. 1.4%) [39]. Thus, these patients will require further management with corticosteroid, immunomodulatory, or cytotoxic agents. Further research into adjunct therapies is an area for future work. Cyclosporine is an example of an immunomodulatory agent that has shown benefit in small observational studies and case reports [40]. Previous studies found that treatment with IVIG alone without aspirin in the acute stage of KD did not affect the response rate of IVIG treatment [16,35]. Although thrombocytosis is common in the subacute stage of KD [5–7], enhanced platelet aggregation was also observed before the initial treatment of KD [41]. Thus, low-dose aspirin is needed for the initial treatment of KD for its antiplatelet effect. In addition, our meta-analysis indicated that the rate of rKD between the low-dose and high-dose aspirin group was not statistically different but was nearly significant with a P-value of 0.05. Therefore, it is difficult to conclude that low-dose aspirin would not affect the risk of rKD. Further, high-dose aspirin had an anti-inflammatory effect, while low-dose aspirin did not. Thus, the first IVIG treatment combined with high-dose aspirin might enhance the anti-inflammatory effect and lead to a lower rate of rKD [42]. However, this hypothesis needs to be further verified by studies with larger sample sizes. While low-dose aspirin could slightly increase the incidence of rKD, it did not increase the rate of CAL, indicating that the efficacy of low-dose aspirin treatment remained acceptable.

Previous studies indicated a shorter duration of fever or hospital stay among the patients receiving high-dose aspirin [43,44], but there were some other studies with inconsistent conclusions [16,35,45]. The present meta-analysis showed no significant difference in the average duration of fever and hospitalization between low-lose and high-dose aspirin groups, indicating that high-dose aspirin treatment had no benefit in reducing the duration of fever or hospitalization compared with low-dose aspirin treatment.

The combination of IVIG 2 g·kg^-1^ and high-dose aspirin (30–50 mg·kg^-1^·d^-1^ or 50–80 mg·kg^-1^·d^-1^) has been recommended for the initial treatment of KD by several guidelines [1,36]. In day-to-day clinical practice, clinicians often use 30–50 mg·kg^-1^·d^-1^ (maximum 4 g·d^-1^) of aspirin, the lower end of the dose range recommended by AHA [14], because higher doses have an increased potential for adverse effects without confirmed benefits. However, the choice of the optimal dose for aspirin remains controversial. In 1995, Durongpisitkul et al. [18] performed a meta-analysis to assess the efficacy of IVIG and aspirin therapy in KD and found no statistically significant difference in the incidence of CAL between the high-IVIG-low-ASA and high-IVIG-high-ASA groups. Aspirin <80 mg·kg^-1^ was defined as the low-dose group in this paper. Two years later, Terai et al. [19] reviewed six randomized, controlled studies that evaluated varying doses of IVIG and aspirin and aspirin alone using blinded echocardiographic assessment to detect CAA and demonstrated no difference in the prevalence of CAA between patients receiving moderate-dose aspirin (30–50 mg·kg^-1^·d^-1^) and those receiving high-dose aspirin (80–120 mg·kg^-1^·d^-1^). These results were consistent with our meta-analysis. However, no studies have assessed the efficacy of aspirin 3–5 mg·kg^-1^·d^-1^ therapy. Furthermore, IVIG dosages were inconsistent in both included studies. Our meta-analysis is the first to assess the efficacy of low-dose aspirin (3–5 mg·kg^-1^·d^-1^) combined with IVIG 2 g·kg^-1^ in the acute phase of KD, which is more consistent with the current treatment regimen [1,36].

## Study limitations

Our study has several limitations. First, the number of included studies was small. Thus, the result of Egger’s publication bias test and the meta-regression analysis should be treated with caution. Second, most studies lacked information about adverse effects, which might interfere with the comparison of efficacy between the two groups. Third, due to high-dose aspirin being widely recommended by guidelines, the low-dose aspirin group was relatively small in most studies, leading to inevitable bias. Additionally, although the current study is not registered and there may be slight deviations, we strictly followed the system evaluation steps. Besides, all of the included studies were nonrandom and retrospective trials, which could lead to biased results. Finally, as none of the studies involved in our meta-analysis used a standard non-inferiority study design, we could not evaluate the non-inferiority of low-dose aspirin in the present study, which should be a more appropriate study design to address this issue.

## Conclusions

In conclusion, treatment with low-dose aspirin compared with high-dose aspirin showed no significant difference in the incidence of CAL, the risk of rKD, or the length of fever or hospital stay in the acute phase of KD. Therefore, low-dose aspirin (3–5 mg·kg^-1^·d^-1^) may be as effective as the high-dose aspirin (≥30 mg·kg^-1^·d^-1^) for the initial treatment of KD. More prospective studies (ideally randomized clinical trials with uniform criteria and a larger number of patients) are needed to evaluate the efficacy of low-dose aspirin for the initial treatment of KD.

## Supporting information

S1 Table PRISMA checklist. (DOC)

S1 Appendix Search strategies for EMBASE, the cochrane central register of controlled trials, china national knowledge infrastructure, and google scholar. (DOCX)
