# Supplementary material for: Efficacy between low and high dose aspirin for the initial treatment of Kawasaki disease: Current evidence based on a meta-analysis
Source: PLoS One. 2019 May 22;14(5):e0217274. doi: 10.1371/journal.pone.0217274 (PMC6531010; doi:10.1371/journal.pone.0217274)
Supplement: S1 Table — (DOC) [file pone.0217274.s001.doc]

| **Section/topic** | **#** | **Checklist item** | **Reported on page #** |
| --- | --- | --- | --- |
| **TITLE** | | |  |
| Title | 1 | Efficacy between low and high dose aspirin for the initial treatment of Kawasaki disease: Current evidence based on a Meta-analysis | 1 |
| **ABSTRACT** | | |  |
| Structured summary | 2 | Kawasaki disease (KD) is now the leading cause of acquired heart disease in children in developed countries. Intravenous immunoglobulin (IVIG) and aspirin were considered as the standard initial treatment of KD for decades. However, the optimal dose of aspirin has remained controversial. In recent years, many studies compared the efficacy of low-dose with high-dose aspirin in the acute phase of KD, but the results have not always been consistent. Therefore, we performed this meta-analysis to evaluate the efficacy of low-dose aspirin compared with high-dose for the initial treatment of KD. Studies related to aspirin therapy for KD were selected from PubMed, EMBASE, the Cochrane Central Register of Controlled Trials, China National Knowledge Infrastructure, and Google scholar through Mar 25th, 2019. Data were analyzed using STATA Version 15.1. Additionally, publication bias and sensitivity analysis were also performed by STATA version 15.1. Six studies were included to be analyzed the rate of coronary artery lesion (CAL), five reports for IVIG-resistant KD (rKD), and four for the duration of fever and hospitalization. However, no significant differences were recorded between low-dose and high-dose aspirin groups in the incidence of CAL (risk ratio (RR), 0.85; 95%CI (0.63, 1.14); P = 0.28), the risk of rKD (RR, 1.39; 95%CI (1.00, 1.93); P = 0.05), and duration of fever and hospitalization (the mean standard deviation (SMD), 0.03; 95%CI (-0.16, 0.22); P = 0.78). Low-dose aspirin (3-5 mg·kg-1·d-1) may be as effective as the use of high-dose aspirin (≥30 mg·kg-1·d-1) for the initial treatment of KD. Besides, further well-designed randomized clinical trials are needed to strictly evaluate the efficacy of low-dose aspirin for the initial treatment of KD. | 1-2 |
| **INTRODUCTION** | | |  |
| Rationale | 3 | Kawasaki disease (KD) is an acute, self-limited febrile vasculitis of unknown cause that predominantly affects children under five years of age. KD is now the most common cause of acquired heart disease in children in developed countries. In general, KD is regarded as an innate immune disorder resulting from the exposure of a genetically predisposed individual to microbe-derived innate immune stimulants. However, the etiology and pathogenesis of KD are still unclear. The degree of platelet ac­tivation was closely associated with the presence of coronary artery complications in the acute stage of KD. Therefore, anti-inflammatory and anti-platelet therapies are the primary treatments for KD. The American Heart Association (AHA) recommends that the standard treatment regimen for the acute phase of KD involves administering IVIG 2 g·kg-1 within ten days of onset and aspirin moderate (30-50 mg·kg-1·d-1) to high-dose (80-100 mg·kg-1·d-1) until the patient is afebrile. This acute phase regimen is designed primarily for anti-inflammatory treatment. However, side effects including gastritis, upper gastrointestinal bleeding, anemia, and Reye’s syndrome have been reported in KD children receiving high-dose aspirin treatment. Previous studies have shown that the incidence of coronary artery lesions (CAL) is highly dependent on the dosage and infusion timing of IVIG, but not related to the aspirin dose. These results suggest that the efficacy of low-dose aspirin may be adequate for the initial treatment of KD, and can also reduce the risk of complications caused by high-dose aspirin. To explore this problem, researchers have recently performed many related studies, but the results have not always been consistent. | 3-4 |
| Objectives | 4 | We performed this meta-analysis to evaluate whether low-dose aspirin was as effective as high-dose aspirin for the initial treatment of KD. | 4 |
| **METHODS** | | |  |
| Protocol and registration | 5 | This analysis was performed following a predetermined protocol by the recommendations of the Cochrane Handbook of Systematic Reviews. The data collection and reporting were in accordance with the Preferred Reporting Items for Systematic Reviews and Meta-Analyses (PRISMA) Statement. | 4-5 |
| Eligibility criteria | 6 | Criteria for inclusion: 1) All subjects were patients diagnosed with KD; 2) randomized controlled or non-randomized controlled clinical trials or cohort studies evaluating the efficacy of low-dose (3-5 mg·kg-1·d-1) and high-dose (≥30 mg·kg-1·d-1) aspirin combined with standard IVIG (2 g·kg-1) therapy for the initial treatment of KD; 3) outcome interests were as follows: the incidence of CAL included CAA, coronary artery dilatation or ectasia, days of fever or hospital, the rate of IVIG-resistant KD (rKD), and the presence of adverse events.  Criteria for Exclusion Studies: meeting any of the following criteria were excluded: 1) patients were not treated with standard IVIG (2 g·kg-1); 2) patients lacked a low-dose or high-dose aspirin group for the initial treatment of KD; 3) conference articles, reviews, or abstracts; 4) duplicate reports. | 5-6 |
| Information sources | 7 | We searched multiple databases including PubMed, EMBASE, the Cochrane Central Register of Controlled Trials, China National Knowledge Infrastructure, and Google scholar through Mar 25th, 2019 to identify relevant studies. | 5 |
| Search | 8 | Keyword search terms were (‘mucocutaneous lymph node syndrome’ OR ‘Kawasaki disease’ OR ‘Kawasaki syndrome’) AND (‘aspirin’ OR ‘acetylsalicylic acid’ OR ‘salicylate’). We searched the PubMed database as follows: (Mucocutaneous Lymph Node Syndrome[MeSH Terms] OR Kawasaki disease OR Kawasaki syndrome) AND (aspirin[MeSH Terms] OR acetylsalicylic acid OR salicylate). Search terms for EMBASE, the Cochrane Central Register of Controlled Trials, China National Knowledge Infrastructure, and Google scholar with corresponding publication numbers can be found in the S1 Appendix. Languages were limited to English and Chinese. | 5 |
| Study selection | 9 | We initially screened studies by title and abstract for the systematic search. A full-text search that retrieved potentially relevant reports was assessed for compliance using the inclusion and exclusion criteria. Inter-rater reliability for the study selection was calculated using the kappa statistic. | 5 |
| Data collection process | 10 | Two investigators (Xiaolan Zheng and Peng Yue) independently assessed the eligibility of reports at the title and abstract level, and a third reviewer (Yifei Li) determining divergence according to the inclusion or exclusion criteria and the quality of the reports. Studies that met all inclusion criteria were selected for further analysis. The baseline data from the included studies were extracted and shown in Table 1. The quality of the included nonunionized studies in meta-analyses was assessed using the Newcastle-Ottawa Scale (NOS) [24]. Additionally, studies which scored at least five stars were considered to have moderate to high methodological quality. | 6 |
| Data items | 11 | Outcome interests were as follows: the incidence of CAL included CAA, coronary artery dilatation or ectasia, days of fever or hospital, the rate of IVIG-resistant KD (rKD), and the presence of adverse events. | 6 |
| Risk of bias in individual studies | 12 | Publication bias was tested using Egger’s regression by Stata statistical software (STATA) version 15.1. Each dot represents each study in the meta-analysis. Asymmetry of the dot distribution between regression lines indicates potential publication bias. A quantified result of P < 0.05 in Egger’s test indicated that publication bias existed. | 7 |
| Summary measures | 13 | The primary outcome was the incidence of CAL, including CAA, coronary artery dilatation or ectasia. Secondary outcomes included rKD, days of hospital or fever, and adverse events. The potential adverse events for aspirin were allergies, digestive symptoms (diarrhea, vomiting and gastric ulcer), abnormal liver function and hemorrhage (skin petechia, epistaxis). | 6 |
| Synthesis of results | 14 | The primary outcome was the incidence of CAL, including CAA, coronary artery dilatation or ectasia. Secondary outcomes included rKD, days of hospital or fever, and adverse events. The potential adverse events for aspirin were allergies, digestive symptoms (diarrhea, vomiting and gastric ulcer), abnormal liver function and hemorrhage (skin petechia, epistaxis) | 6 |

Page 1 of 2

| **Section/topic** | **#** | **Checklist item** | **Reported on page #** |
| --- | --- | --- | --- |
| Risk of bias across studies | 15 | Publication bias was tested using Egger’s regression by Stata statistical software (STATA) version 15.1. Each dot represents each study in the meta-analysis. Asymmetry of the dot distribution between regression lines indicates potential publication bias. A quantified result of P < 0.05 in Egger’s test indicated that publication bias existed. | 6 |
| Additional analyses | 16 | We performed the meta-regression to detect where the potential factor for heterogeneity originated. Sensitivity analysis was conducted for every study to determine whether any single study incurred undue weight in the analysis using STATA 15.1 for meta-analysis fixed/random-effects estimates. | 7 |
| RESULTS | | |  |
| Study selection | 17 | Initially, 2577 potentially relevant papers were retrieved by the aforementioned search method, of which, 31 articles were considered to be of interest after reading titles and abstracts. However, 25 articles were excluded by reading the complete articles due to article type (n = 7), not using the standard IVIG dose (2 g·kg-1) during the initial treatment (n = 13), and lacking a low-dose aspirin (3-5 mg·kg-1·d-1) group for the initial treatment of KD (n = 5). Ultimately, six English reports were included in the meta-analysis, while no Chinese reports met the inclusion criteria. | 8 |
| Study characteristics | 18 | The characteristics of the involved patients in all included studies were presented in Table 1. The six published reports enrolled a total of 11,103 children, of which were 1,753 low-dose aspirin cases. Six studies that were included came from five countries. All included studies were nonrandom and retrospective trials. Two studies did not specify a criteria for dividing patients into a low-dose or high-dose aspirin group, three studies grouped patients by different centers, and one study chose different doses of aspirin depending on the inflammatory level and doctors’ opinions. Furthermore, three studies did not mention the diagnostic criteria for KD, while another three reports used the AHA diagnostic criteria. Moreover, one study scored NOS five stars and was considered to be moderate quality, while the other five reports were awarded ≥ six stars and qualified as high quality. | 8 |
| Risk of bias within studies | 19 | The Egger’s publication bias plot did not show marked asymmetry. | 9-10 |
| Results of individual studies | 20 | Six studies were included to be analyzed the rate of coronary artery lesion (CAL), five reports for IVIG-resistant KD (rKD), and four for the duration of fever and hospitalization. However, no significant differences were recorded between low-dose and high-dose aspirin groups in the incidence of CAL (risk ratio (RR), 0.85; 95%CI (0.63, 1.14); P = 0.28), the risk of rKD (RR, 1.39; 95%CI (1.00, 1.93); P = 0.05), and duration of fever and hospitalization (the mean standard deviation (SMD), 0.03; 95%CI (-0.16, 0.22); P = 0.78). | 9-10 |
| Synthesis of results | 21 | Six studies were included to be analyzed the rate of coronary artery lesion (CAL), five reports for IVIG-resistant KD (rKD), and four for the duration of fever and hospitalization. However, no significant differences were recorded between low-dose and high-dose aspirin groups in the incidence of CAL (risk ratio (RR), 0.85; 95%CI (0.63, 1.14); P = 0.28), the risk of rKD (RR, 1.39; 95%CI (1.00, 1.93); P = 0.05), and duration of fever and hospitalization (the mean standard deviation (SMD), 0.03; 95%CI (-0.16, 0.22); P = 0.78). | 9-10 |
| Risk of bias across studies | 22 | The Egger’s publication bias plot did not show marked asymmetry. | 9-10 |
| Additional analysis | 23 | We performed the meta-regression analysis to identify the potential factors that might cause heterogeneity. All potential factors that were extracted from the baseline measurements and original testing procedures were taken into consideration for the meta-regression. The meta-regression could determine the correlation between the potential factors and the existing heterogeneity. When a significant difference was discovered, the factor should have a dramatic impact on the homogeneity of the enrolled studies with a P value < 0.05. The study regions, the diagnostic criteria for KD, and the specific dosages of the high-dose aspirin groups were taken into account in the meta-regression to detect the origins of heterogeneity. According to the results, the meta-regression did not detect that the study regions had a dramatic impact on the homogeneity of the enrolled studies, P = 0.220, t = 1.45, 95%CI (0.87, 1.55). The meta-regression did not find that the diagnostic criteria for KD was a dramatic impact factor, P = 0.542, t = 0.67, 95%CI (0.43, 4.02). The meta-regression also did not detect that specific dosages from the high-dose aspirin groups had a dramatic impact on the homogeneity of the enrolled studies, P = 0.713, t = -0.39, 95%CI (0.35, 2.19. Therefore, different regions, different diagnostic criteria for KD, and the differences in specific dosage in the high-dose aspirin group were not responsible for the existing heterogeneity. Furthermore, we systematically and qualitatively analyzed the sensitivity across the included studies to determine the influence of individual trials on the results. Finally, we did not detect any significant impact from any single study and confirmed the direction of the results. | 10-11 |
| DISCUSSION | | |  |
| Summary of evidence | 24 | This result suggested that treatment with low-dose aspirin compared with high-dose aspirin showed no significant difference in the incidence of CAL, the risk of rKD, or the length of fever or hospital stay in the acute phase of KD. | 11-14 |
| Limitations | 25 | Our study has several limitations. First, the number of included studies was small. Thus, the result of Egger’s publication bias test and the meta-regression analysis should be treated with caution. Second, most studies lacked information about adverse effects, which might interfere with the comparison of efficacy between the two groups. Third, due to high-dose aspirin being widely recommended by guidelines, the low-dose aspirin group was relatively small in most studies, leading to inevitable bias. Additionally, although the current study is not registered and there may be slight deviations, we strictly followed the system evaluation steps. Besides, all of the included studies were nonrandom and retrospective trials, which could lead to biased results. Finally, as none of the studies involved in our meta-analysis used a standard non-inferiority study design. So that, we could not evaluate the non-inferiority of low-dose aspirin in the present study, which should be a more appropriate study design to address this issue. | 14 |
| Conclusions | 26 | In conclusion, treatment with low-dose aspirin compared with high-dose aspirin showed no significant difference in the incidence of CAL, the risk of rKD, or the length of fever or hospital stay in the acute phase of KD. Therefore, low-dose aspirin (3-5 mg·kg-1·d-1) may be as effective as the high-dose aspirin (≥30 mg·kg-1·d-1) for the initial treatment of KD. More prospective studies with a larger number of patients who ideally are randomized clinical trials with uniform criteria are needed to evaluate the efficacy of low-dose aspirin for the initial treatment of KD. | 14-15 |
| FUNDING | | |  |
| Funding | 27 | This work was supported by grants from the National Natural Science Foundation of China (No. 81700360, 81741025, 81570369 and 81571515) and the Technology Project of Sichuan Province of China (2016SZ0056). |  |

*From:*  Moher D, Liberati A, Tetzlaff J, Altman DG, The PRISMA Group (2009). Preferred Reporting Items for Systematic Reviews and Meta-Analyses: The PRISMA Statement. PLoS Med 6(7): e1000097. doi:10.1371/journal.pmed1000097

For more information, visit: **www.prisma-statement.org**.

Page 2 of 2
