# Supplementary material for: Efficacy between low and high dose aspirin for the initial treatment of Kawasaki disease: Current evidence based on a meta-analysis
Source: PLoS One. 2019 May 22;14(5):e0217274. doi: 10.1371/journal.pone.0217274 (PMC6531010; doi:10.1371/journal.pone.0217274)
Supplement: S1 Appendix — (DOCX) [file pone.0217274.s002.docx]

Search strategies for EMBASE, the Cochrane Central Register of Controlled Trials, and China National Knowledge Infrastructure.

A: Embase database was searched as follows: #1 'lupus erythematosus, systemic' OR 'systemic lupus erythematosus' OR '[lupus](javascript:;) erythematosus' OR '[lupus](javascript:;) [nephritis](javascript:;)', #2 'MicroRNAs' OR 'MicroRNA' OR 'miRNAs' OR 'miRNA', #1 AND #2.

| Embase | | |
| --- | --- | --- |
| 1 | ('lupus erythematosus, systemic' or 'systemic lupus erythematosus' or 'lupus erythematosus' or 'lupus nephritis').mp. [mp=title, abstract, heading word, drug trade name, original title, device manufacturer, drug manufacturer, device trade name, keyword, floating subheading word, candidate term word] | 107165 |
| 2 | ('MicroRNAs' or 'MicroRNA' or 'miRNAs' or 'miRNA').mp. [mp=title, abstract, heading word, drug trade name, original title, device manufacturer, drug manufacturer, device trade name, keyword, floating subheading word, candidate term word] | 133842 |
| 3 | #1 AND #2 | 871 |

## B: the Cochrane Central Register of Controlled Trials database was searched as follows:  (‘lupus erythematosus, systemic’ OR ‘systemic lupus erythematosus’ OR ‘lupus erythematosus’ OR ‘[lupus](javascript:;) [nephritis](javascript:;)’) AND (‘MicroRNAs’ OR ‘MicroRNA’ OR ‘miRNAs’ OR ‘miRNA’) in Title Abstract Keyword

| the Cochrane Central Register of Controlled Trials | | |
| --- | --- | --- |
| 1 | (‘lupus erythematosus, systemic’ OR ‘systemic lupus erythematosus’ OR ‘lupus erythematosus’ OR ‘[lupus](javascript:;) [nephritis](javascript:;)’) in Title Abstract Keyword | 2013 |
| 2 | (‘MicroRNAs’ OR ‘MicroRNA’ OR ‘miRNAs’ OR ‘miRNA’) in Title Abstract Keyword | 716 |
| 3 | #1 AND #2 | 2 |

## C: China National Knowledge Infrastructure database was searched as follows:  (mucocutaneous lymph node syndrome OR Kawasaki disease OR Kawasaki syndrome) AND (MicroRNAs OR MicroRNA OR miRNAs OR miRNA) in Title Abstract Keyword.

| China National Knowledge Infrastructure database | | |
| --- | --- | --- |
| 1 | (lupus erythematosus, systemic OR systemic lupus erythematosus OR lupus erythematosus OR [lupus](javascript:;) [nephritis](javascript:;)) in Title Abstract Keyword | 37985 |
| 2 | (MicroRNAs OR MicroRNA OR miRNAs OR miRNA) in Title Abstract Keyword | 41185 |
| 3 | #1 AND #2 | 26 |
